# Different Roles of Tocopherols and Tocotrienols in Chemoprevention and Treatment of Prostate Cancer

**DOI:** 10.1016/j.advnut.2024.100240

**Published:** 2024-05-10

**Authors:** Qing Jiang

**Affiliations:** Department of Nutrition Science, Purdue University, West Lafayette, IN, United States

**Keywords:** long-chain carboxychromanol, 5-lipoxygenase, inflammation, tocopherol, prostate cancer, tocotrienol, medicine

## Abstract

The vitamin E family contains α-tocopherol (αT), βT, γT, and δT and α-tocotrienol (TE), βTE, γTE, and δTE. Research has revealed distinct roles of these vitamin E forms in prostate cancer (PCa). The ATBC trial showed that αT at a modest dose significantly decreased PCa mortality among heavy smokers. However, other randomized controlled trials including the Selenium and Vitamin E Cancer Prevention Trial (SELECT) indicate that supplementation of high-dose αT (≥400 IU) does not prevent PCa among nonsmokers. Preclinical cell and animal studies also do not support chemopreventive roles of high-dose αT and offer explanations for increased incidence of early-stage PCa reported in the SELECT. In contrast, accumulating animal studies have demonstrated that γT, δT, γTE, and δTE appear to be effective for preventing early-stage PCa from progression to adenocarcinoma in various PCa models. Existing evidence also support therapeutic roles of γTE and its related combinations against advanced PCa. Mechanistic and cell-based studies show that different forms of vitamin E display varied efficacy, that is, δTE ≥ γTE > δT ≥ γT >> αT, in inhibiting cancer hallmarks and enabling characteristics, including uncontrolled cell proliferation, angiogenesis, and inflammation possibly via blocking 5-lipoxygenase, nuclear factor κB, hypoxia-inducible factor-1α, modulating sphingolipids, and targeting PCa stem cells. Overall, existing evidence suggests that modest αT supplement may be beneficial to smokers and γT, δT, γTE, and δTE are promising agents for PCa prevention for modest-risk to relatively high-risk population. Despite encouraging preclinical evidence, clinical research testing γT, δT, γTE, and δTE for PCa prevention is sparse and should be considered.

**Statements of significance**

This article is the first comprehensive and critical review assessing distinct roles of α-tocopherol and other members in the vitamin E family in chemoprevention and therapy of prostate cancer, which is the most common and second most lethal cancer in males.

## Introduction

Prostate cancer (PCa) is the most common cancer and the second most lethal cancer in males [1]. Since 2014, the incidence rate of PCa has increased by 3% per year overall and by ∼5% per year for advanced-stage PCa, according to the American Cancer Society. Although androgen deprivation therapy is effective for treating locally advanced or metastatic PCa, the cancer may eventually relapse and progress to metastatic castration-resistant prostate cancer (CRPC). CRPC is associated with poor prognoses and has the mean survival time of only 16–18 mo [2,3]. There is no effective treatment for advanced-stage cancer. The lack of effective therapies for late-stage cancer underscores the importance of early detection and intervention for reducing PCa mortality [4,5]. Although primary prevention emphasizes healthy life styles among general population with no increased risks, secondary and tertiary prevention includes chemoprevention using natural and synthetic compounds to inhibit progression of early-stage cancer to advanced disease among moderate-risk or high-risk population, for example, those with driver mutations or early-stage cancer [6]. Many efforts have been made to search for effective and safe chemopreventive agents for PCa prevention including vitamin E. Natural forms of vitamin E consist of 8 lipophilic antioxidants, including α-tocopherol (αT), βT, γT, and δT and α-tocotrienol (TE), βTE, γTE, and δTE. Research on vitamin E and PCa was initially focused on αT in cell, animal, and epidemiologic/population studies. Noticeably, some large randomized controlled trials (RCTs) have also been carried out to evaluate the effect of αT supplementation on PCa. Subsequently, potential effects of other forms of vitamin E such as γT, δT, γTE, and δTE on PCa have also been assessed in preclinical cell and animal studies. Although the roles of tocopherols and TEs in cancer prevention have been reviewed elsewhere [[7], [8], [9], [10]], it is worth assessing distinctive functions of these compounds for secondary and tertiary chemoprevention of PCa and even therapy against advanced PCa. In this article, I focus on current knowledge of αT and other members, that is, γT, δT, γTE and δTE, with respect to their roles in PCa prevention and treatment via critically reviewing cell-based and mechanistic studies, preclinical research in PCa animal models, and RCTs, without including epidemiologic data that have been recently summarized in a systematic review [11].

## Current Status of Knowledge

### Different forms of vitamin E and bioavailability

**FIGURE 1:**
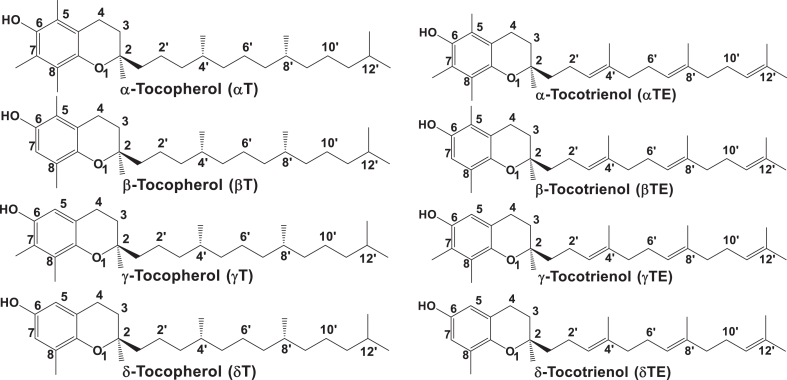
Chemical structures of natural forms of vitamin E.

The structures of all vitamin E isoforms share a chromanol ring with a phenolic group at the 6-position, which offers antioxidant activities capable of terminating lipid radical chain reactions. Tocopherols have a phytyl side chain, whereas TEs have a similar side chain with 3 double bonds (Figure 1) [12]. The α, β, γ, and δ isoforms differ at the 5 or 7 positions occupied with either a hydrogen or methyl group (Figure 1). Vitamin E forms are naturally synthesized by plants and mainly found in seeds, nuts, and plant oils [[12], [13], [14], [15]]. For instance, αT is the predominate vitamin E in almonds, peanuts, and sunflower seeds, and γT and δT are the main vitamin E in walnuts, pecan, and sesamin seeds. γTE is rich in barley and palm oil, and δTE is mainly found in annatto plants.

Although all vitamin E forms are believed to have similar antioxidant activities, these compounds have greatly different bioavailability. For instance, αT is the major form of vitamin E in the blood and tissues and considered the bona fide vitamin E as deficiency of αT results in vitamin E deficiency–associated ataxia [16]. Serum concentrations of αT in humans have been reported ranging from ∼20 μM to >50 μM varying with intake doses and other dietary components such as fat contents [17]. The half-life (T_1/2_ = time needed from C_max_ to 50% C_max_) of αT in response to supplementation has been documented to be ∼30 h [17]. γT is often the second abundant vitamin E found in tissues with serum concentrations of γT at 1–3 when γT is provided from foods. On supplementation, γT may transiently reach to 20–30 μM or even higher [[18], [19], [20]] with T_1/2_ of 6–8 h [21]. Although relatively low in the plasma, γT appears to be accumulated in peripheral tissues including adipose and muscle with concentrations of 176 and 107 nmol/g, respectively, as documented by Burton et al. [22]. Two-week supplementation of γT-rich tocopherols has recently been reported to result in an increase of γT and δT in the prostate of patients undergoing radical prostatectomy [23]. In response to oral supplementation of 200–3200 mg γTE and δTE, these TEs appeared to transiently increase in the blood to C_max_ of 5–10 μM with half-time of 2–4 h [24,25]. In summary, based on above-cited literature, vitamin E forms have highly varied bioavailability with the order of αT >> γT >> γTE, δTE, and this order is inversely associated with their relative degree of being catabolized in vivo, as discussed in the next section.

### Vitamin E metabolism and metabolites

**FIGURE 2:**
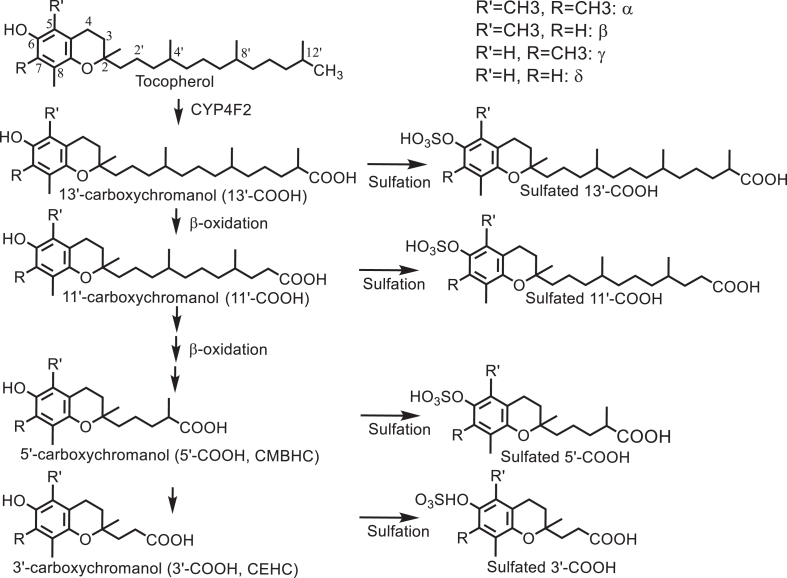
Vitamin E (tocopherols as the example) metabolism: tocopherols are metabolized by CYP4F2-initiated side-chain oxidation to form various carboxychromanols and sulfated carboxychromanols. CEHC (3'-COOH), carboxyethyl-hydroxychroman; CMBHC (5'-COOH), carboxymethylbutyl hydroxychroman.

As extensively discussed in previous reviews [12,26], varied bioavailability of vitamin E forms is regulated by their differential affinity to α-tocopherol transport protein (TTP) and different degree of catabolism. In particular, high bioavailability of αT in tissues is a result of its high-affinity binding to TTP, which facilitates αT to be incorporated into VLDLs and prevents it from being catabolized in the liver [12,26]. Consequently, only a small portion of αT (<1%) is metabolized [26]. In contrast, other vitamin E forms have much lower affinity to TTP than αT and are readily catabolized via cytochrome P-450 (CYP; CYP4F2 or murine *Cyp4f14*)-catalyzed side chain hydroxylation and oxidation of the terminal carbon 13' in the endoplasmic reticule to form 13'-hydroxychromanol (13'-OH) and 13'-carboxychromanol (13'-COOH) (Figure 2). CYP4F2 has been shown to have differential catabolic activities toward different forms of vitamin E with the order of δTE, γTE > δT, and γT >> αT [26]. 13'-COOHs are further metabolized through β-oxidation to intermediate-chain and short-chain carboxychromanols including 11'-,9'-,7'-,5'-COOH (also called carboxymethylbutyl hydroxychromans) and 3'-COOH [also called 3'-carboxyethyl-hydroxychroman (CEHC)] (Figure 2). Conjugated metabolites including sulfated long-chain carboxychromanols and sulfated CEHCs are detected in human cells and in the plasma of rodents, indicating that sulfation takes place parallel to side chain oxidation [27,28].

The bioavailability of different vitamin E metabolites has also been documented in humans and rodents. For instance, γ-CEHC may transiently reach to ≤15 μM in the serum of humans as a result of γT supplementation [[18], [19], [20],^29^]. Supplementation of δTE at 400 to 3200 mg/d has been shown to result in an increase of δ-CEHC to ≤10–15 μM and 5'-COOH (δ-carboxymethylbutyl hydroxychromans) to 4–5 μM in the plasma and excretion of δ-CEHC (≤∼91 μM) in the urine [30]. As to long-chain metabolites, sulfated 11'-COOHs have been reported in the circulation in rodents supplemented with tocopherols and TEs [28]. 13'-COOHs, which are low in the circulation, appear to be the predominant metabolites in feces of rodents fed with tocopherols or TEs [28,[31], [32], [33], [34]]. These bioavailability data of metabolites are useful for assessing potential contribution of metabolism to the activities of different forms of vitamin E in vivo, especially considering that CEHCs and 13'-COOHs have been shown to have anticancer and anti-inflammatory effects (see further sections).

### αT and PCa

αT is the only vitamin E that has been investigated for cancer prevention in large RCTs. In this section, I focus on RCTs that assessed the impact of αT supplementation on PCa as either the primary or a secondary end point and analyze key differences of these studies concerning αT’s dosing, participants’ characteristics, and disease stages (Table 1) [[35], [36], [37], [38], [39], [40], [41]]. Moreover, I will correlate RCT data with evidence from preclinical cell and animal studies concerning the role of supplementation of high-dose αT in PCa prevention.

**TABLE 1:** The effects of supplementation of α-tocopherol on PCa (primary or secondary end point) in randomized, placebo-controlled intervention trials

Study and duration	Characteristics of participants	Intervention	Major end points/outcomes
The α-Tocopherol, β-Carotene Cancer Prevention Study (ATBC), Southwestern, Finland [35]: 5–8 y (1985 to 1993)	29,133 men, 50–69 y, all heavy smokers, healthy (no previous cancer or other serious diseases)	50 mg/d *dl*-α-tocopheryl acetate (*dl*-α-TA), 20 mg/d β-carotene, or their combination	1': Lung cancer. 2': Prostate, bladder, colon and rectum, stomach cancer. Outcomes: *dl*-α-TA ↓ PCa death; ↑deaths from hemorrhagic stroke
Heart Protection Study MRC/BHF (HPS), United Kingdom [36]; 5 y (1994–2001)	20,536 individuals, 40–80 y with coronary disease, other occlusive arterial disease, or diabetes	600 mg synthetic vitamin E, 250 mg vitamin C, 20 mg β-carotene daily	1': Coronary events and fatal or nonfatal vascular events 2': Cancer and other major morbidity Outcomes: no benefit
The Heart Outcomes Prevention Evaluation (HOPE) and The Ongoing Outcomes (HOPE-TOO), multi-European countries and United States [37]; median 7 y (1993–2003)	7030 patients aged 55 y or older with vascular disease or diabetes mellitus from the initial HOPE trial (1993–1999) and the HOPE-TOO extension (1999–2003)	400 IU/d RRR-α-tocopherol acetate	1': Cancer incidence, cancer-related deaths, and major cardiovascular events Outcomes: no benefits (including PCa incidence) and ↑ rates of heart failure
Physician’s Health Study II (PHS-II), United States [38]; 8 y (1997–2007)	14,641 physicians, men aged 50 y or older including 1307 men with a history of cancer including PCa	400 IU synthetic α-tocopherol every other day and vitamin C 500 mg daily	1': PCa for vitamin E and total cancer for vitamin C 2': Total cancer for vitamin E Outcomes: no effects on prostate or total cancer incidence or death
Progression from HGPIN to PCa, Canada [39]; 3 y of supplements plus 3-y follow-up (2003–2010)	303 patients (the median age of 62.8 y), confirmed with HGPIN, median PSA ∼5.41 ng/mL	Soy (40 g), *dl*-α-tocopherol (800 IU), selenium (200 μg) daily vs. placebo	1': Progression from HGPIN to invasive PCa Outcomes: supplementation did not show beneficial effects
Selenium and Vitamin E Cancer Prevention Trial (SELECT), Canada, Puerto Rico, United States [40,41]; 7–12 y (2001–2011)	35,533 men, 50 y or older for African American and 55 y or older among other races. PSA ≤ 4 ng/mL and not diagnosed or suspicious for PCa based on DRE screening	400 mg IU *dl*-α-TA, 200 μg/d L-selenomethionine, or their combination	1': PCa. 2': Lung, colorectal, and overall primary cancer. Outcomes: ↑ incidence of early-stage PCa; no effects on other types of cancer

Abbreviations: 1', primary end point; 2', secondary end point; DRE, digital rectal examination; HGPIN, high-grade prostatic intraepithelial neoplasia; PCa, prostate cancer; RCT, randomized controlled trial; Se, selenium; ↓, suppress or inhibit; ↑, increase or enhance.

#### The impact of αT supplement on PCa in randomized, double-blinded, and placebo-controlled intervention studies

From 1985 to 1993, the Alpha-Tocopherol, Beta-Carotene Cancer Prevention (ATBC) study was carried out in Finland to evaluate the effect of daily supplementation of αT, β-carotene, or their combination on the incidence of lung (the primary outcome) and other cancers including PCa (secondary outcomes) in male heavy smokers smoking 20 cigarettes/d [35]. Randomly assigned participants received 50 mg/d *dl*-α-tocopheryl acetate (*n* = 7286), 20 mg/d β-carotene (*n* = 7282), or both compounds (*n* = 7278) and placebo (*n* = 7287) capsules. Compared with the placebo controls, αT did not show a significant effect on the incidence of lung cancer, whereas β-carotene unexpectedly increased the risk of lung cancer and total mortality [35]. Interestingly, participants receiving αT had 32% (95% CI: −47%, −12%) and 41% (95% CI: −65%, −1%) reduction of PCa incidence and mortality (*P* < 0.05), respectively, compared with those not receiving it [35,42]. It is also noteworthy that αT supplementation appeared to decrease the incidence of clinical PCa (stage II–IV) but not stage 0–I [42] while had more death from hemorrhagic stroke when compared with no-αT group. In the 6–8 y ATBC postintervention follow-up study, no significant beneficial effect of αT was observed on posttrial PCa incidence [43]. However, in the 18-y ATBC postintervention follow-up study, Virtamo et al. [44] observed that although neither αT nor β-carotene supplement had significant effects on posttrial cancer incidence, the preventive effect of αT on PCa appeared to continue as αT decreased posttrial PCa mortality (RR: 0.84; 95% CI: 0.7, 0.99).

Given the promising data observed from ATBC trial, the SELECT was conducted to evaluate the effects of αT (400 IU/d), selenium (200 μg/d) or their combination on PCa from 2001 to 2011 [40]. In the SELECT, participants mainly included healthy nonsmoking men (92% not current smokers) who did not have elevated prostate-specific antigen (PSA, ≤4 ng/mL) or PCa as assessed by digital rectal examinations (DREs). After 5.46-y supplementation, >95% diagnosed PCa was in early stages, for example, ∼70% and 25% in stage T1 and T2, respectively, and there was a nonsignificant (*P* = 0.06) increase in stage I PCa in αT-alone group [40]. After the initial publication in 2009, participants’ follow-up continued, and by 2011, additional cases of PCa were diagnosed. At this time, compared with placebo, the hazard ratios concerning PCa in the αT, selenium, and αT+Se groups were estimated to be 1.17 (99% CI: 1.004, 1.36; *P* = 0.008), 1.09 (99% CI: 0.93, 1.27; *P* = 0.18), and 1.05 (99% CI: 0.89, 1.22; *P* = 0.46), respectively [41]. All identified PCa cases were at early stages, that is, 70%–75% stage I (clinically inapparent) and 25%–30% stage II (clinical cancer with tumor confined in prostate). It was therefore concluded that αT supplement increased the risk of early-stage PCa.

In addition to the SELECT, 4 additional RCTs were conducted between 1993 and 2011 aiming to examine the effects of αT (400 IU) on the incidence of cardiovascular diseases and cancer including PCa, that is, MRC/BHF (HPS) [36], HOPE/HOPE-TOO [37], PHS-II [38], and Canadian high-grade prostatic intraepithelial neoplasia (PIN) progression to invasive cancer study [39]. Like the SELECT, these studies did not observe beneficial effects of αT (400–800 IU) on cancers including PCa (Table 1) [[35], [36], [37], [38], [39], [40], [41]].

#### Key differences between ATBC and other RCT trials

Although all above-mentioned RCT trials (Table 1) [[35], [36], [37], [38], [39], [40], [41]] are randomized and placebo-controlled intervention studies assessing potential preventive effects of αT supplement on cancer including PCa, there are some key differences between the ATBC and other trials including the SELECT. First, all participants in the ATBC study were heavy smokers, whereas low percentage of participants in other RCTs are heavy smokers. For instance, the SELECT had <8% current smokers. Second, the ATBC trial tested modest supplementation of αT (50 mg), whereas the SELECT and other trials used high supplements of αT at 400 IU or higher. Third, unlike the SELECT, there was no comprehensive pretrial screening for PSA or DRE in the ATBC trial. Additionally, the ATBC trial reported that αT decreased the incidence of clinical (stage II–IV) PCa and PCa-associated mortality, but not stage 0–I PCa, whereas the SELECT exclusively focused on the impact on early-stage PCa (>70% latent and 25%–30% stage II PCa) incidence. Because of these distinct features including αT dosing, smoking status of participants, and PCa stage, it is hardly surprising that the ATBC trial revealed different outcomes from other studies concerning the role of αT in PCa.

It is noteworthy that the lack of PSA and DRE screening, together with PCa as a secondary end point, may be potential sources of biases for the ATBC trial. However, the number of participants diagnosed with prostatic hyperplasia between αT and non-αT groups was similar in the ATBC trial. Further, there was no difference between αT and non-αT groups with respect to the increase in serum PSA [42]. Moreover, the observation that αT supplementation decreased PCa-associated mortality in the ATBC trial also suggests a true effect of αT. For these reasons, the result from the ATBC trial that moderate supplementation of αT appears to be beneficial to heavy smokers for preventing clinical PCa is important and should be paid attention to.

#### Preventive effects of high doses of αT on PCa in preclinical animal studies

From early 1990s, the role of αT or its combinations with other antioxidants in PCa prevention has been evaluated in animal models including carcinogens-induced PCa and genetically engineered spontaneous tumor-developing models. In 1991, Nakamura et al. [45] studied the effect of the αT (1% diet)-supplemented diet together with other antioxidants on 3,2'-dimethyl-4-aminobifenyl–induced prostate carcinogenesis in rats but did not observe beneficial effects on the incidence of tumors in the prostate or any other organs analyzed [7]. In another study, Ozten et al. [46] reported that *dl*-αT at 0.2% and 0.4% in diet failed to block PCa stimulated by testosterone plus estradiol in NBL rats. Similarly, αT at 2% and 4% or its combinations with selenomethionine did not decrease the incidence of PCa induced by sequential administration of cyproterone acetate, testosterone propionate, and N-methyl-N-nitrosourea as well as chronic androgen stimulation with subcutaneous implantation of testosterone capsules in rats [47]. In addition, the effects of αT on PCa have been assessed along with other forms of vitamin E in different PCa models including phosphatase and tensin homolog deleted on chromosome 10 (PTEN) knockout (Pten^P−/−^) and transgenic rat for adenocarcinoma of prostate (TRAP) mice [48,49]. Most of these studies revealed that αT at doses ranging from 0.05% to 0.2% was ineffective for inhibition of PCa development (Table 2) [[45], [46], [47], [48], [49], [50], [51], [52], [53], [54], [55], [56], [57], [58], [59], [60], [61], [62], [63], [64], [65]].

**TABLE 2:** Chemopreventive and potential therapeutic effects of tocopherols and tocotrienols in preclinical PCa models

Time	Vitamin E forms and doses	PCa models	Key observations
Chemopreventive effects on carcinogen-induced PCa in rodents			
1991	1% αT along with catechol (0.8%), resorcinol (0.8%), hydroquinone (0.8%), selenium (2 ppm), γ-orysanol (2%) for 40 wk [45]	DMAB-induced prostate carcinogenesis in male F344 rats	No significant effects on atypical hyperplasias and carcinomas of the prostate
2010	*dl*-αT at 2000 and 4000 mg/kg diet (equivalent to 0.2% and 0.4% diet), or L-selenomethionine (1.5 or 3 mg/kg) [46]	Testosterone plus estradiol-treated NBL rats	No effects on development of prostate carcinomas, whereas αT ↑ the incidence of adenocarcinomas of the mammary glands
2010	*dl*-αT at 2000 and 4000 mg/kg diet, or αT (500 or 2000 mg/kg) plus L-selenomethionine (3 mg/kg) for 13 mo [47]	Intravenous injection of MNU plus androgen stimulation in Wistar-Unilever (W/U) rats	No significant effects on PCa incidence or development
2013	γT-enriched diet (20 mg/kg) for 16 wk [50]	MNU plus testosterone (subcutaneously)-induced epithelial dysplasia in the ventral prostate of Wistar rats	γT ↓ MNU-induced epithelial dysplasia by 38%, epithelial proliferation, COX-2, and MMP-9 in the ventral prostate
2016	γTmT (0.3%) or individual tocopherols, i.e., γT, δT, or αT at 0.2% in diet [51]	PhIP-induced prostate carcinogenesis in the CYP1A-humanized (hCYP1A) mice	γTmT and individual tocopherols ↓ PhIP-induced mouse PINs by 66%. δT (↓60% PINs) is stronger than γT or αT (↓∼20% PINs) for this effect
Chemopreventive effects on PIN development or PIN to adenocarcinoma in genetically engineered models			
2009	γT at 50-mg/kg, 100-mg/kg, and 200-mg/kg diet for 7–10 wk; αT at 50-mg/kg diet [49]	The transgenic rat for adenocarcinoma of prostate (TRAP) driven by probasin/SV40 T antigen	γT, in contrast to αT, ↓ multiplicity of prostate adenocarcinoma without affecting PIN, and ↑ caspase 3 and caspase 7 in prostate tissue
2009, 2012	γTmT at 0.1% in diet for 24 wk [52,53]	Oncogenic SV40-driven transgenic andenocarcinoma of mouse prostate (TRAMP) mice	γTmT ↓ palpable tumor incidence by 75% and ↓ % high-degrade PINs, and ↑Nrf2 and its targeted genes by ↓CpG methylation
2010	γTE-rich tocotrienols (TEs) at 0.1%, 0.3%, and 1% containing αTE, βTE, γTE, δTE, and αT at 13, 1, 19, 5, and 13%, respectively, in AIN-76A diet [54]	TRAMP mice	TEs dose-dependently ↓ tumor incidence (50%–70%), weight (by 75%) and high-grade neoplastic lesions, whereas ↑ BAD, caspase 3, p21, and p27
2018	δT or αT at 0.2% in diet from the age of 6 or 12–40 wk [48]	Male prostate-specific PTEN-knockout (Pten^P−/−^) mice	δT (intervention starting at 6 or 12 wk of age), but not αT, ↓ % and multiplicity of prostate adenocarcinoma by 43%–53%, p-AKT and Ki67, and ↑caspase 3, without affecting HGPINs
2022	δTE at 0.05% diet from age of 6 wk to 20 to 40 wk [55]	Male prostate-specific PTEN-knockout (Pten^P−/−^) mice	δTE ↓ % prostate adenocarcinoma by 32.7%, proliferation (Ki67), angiogenesis (CD31 and VEGFd), and ↑apoptosis (caspase 3)
Effects on the growth of relatively advanced PCa			
2004	540 ppm αT (originally from Rovimix E 50 from DSM), 200 ppm lycopene, or their combination for 4-wk pretreatment and 18-d posttumor implantation [56]	1 × 10^5^ MatLyLu Dunning prostate cells were injected into the ventral prostate of male Copenhagen rats	αT or lycopene but not their combination ↑ % of necrotic area in a cross-sectional view of prostate tumor tissue; αT ↓ androgen signaling and lycopene ↓ 5-α-reductase, both ↓ prostatic spermine–binding protein
2006	*dl*-αT acetate at 5 or 50 mg/kg BW, or lycopene at 5 or 50 mg/kg BW, or lycopene+αT (5 mg/kg BW each) [57]	Orthotopic PC-346C prostate xenograft model in NMRI nu/nu mice	Lycopene+αT but not either agent alone suppressed orthotopic growth of PC-346C prostate tumors by 73% and increased median survival time by 40%
2006	γTE at 400 mg/kg BW was injected subcutaneously in the neck of nude mice, which were then irradiated at the rear part of the body including the location of tumor [58]	Human PCa bone metastasizing PC3 cells implanted in athymic mice	The size of the tumors was ↓ by ∼40% only in γTE-injected and γTE-irradiated mice, whereas there was ↑ lipid peroxidation in tumors and kidney (potential side effect at kidney)
2010	γT at 200 mg/kg or its combination with lycopene (250 mg/kg diet) [59]	Dunning R3327H adenocarcinoma cells implanted in male Copenhagen rats	Neither γT nor its combination with lycopene had significant impact on tumor growth
2010	γTE at 50 mg/kg via intraperitoneally 5 times a week alone or coadministered with docetaxel (7.5 mg/kg via intraperitoneally) [60]	PC-3 human androgen-independent PCa in xenograft model	γTE and γTE+docetaxel ↓ tumor growth by 52% and 61%, respectively. γTE was accumulated in tumors, ↑apoptosis, and ↓ proliferation
2011	γT or γTE at 125 mg/kg BW by oral gavage 3 times a week for 5 wk [61]	Human LNCaP PCa xenograft model in nu/nu mice	γTE was stronger than γT in ↓ the growth of LNCaP xenograft (by 50%) min nude mice
2011	γTE (100 mg/kg daily) orally for 3 wk, and PCa cells were subcutaneously injected for another 4 wk; γTE-pretreated PCa cells injected subcutaneously or orthotopically in nude mice [62]	PC3-Luc cells subcutaneously or orthotopically injected in nude mice	γTE-pretreated cells resulted in ↓tumor incidence; γTE given orally also ↓tumor incidence
2014	Methaneseleninic acid (MSA) (40.95 μg/kg BW), γT1 (20.83 mg/kg) or γT2 (41.66 mg/kg BW) in corn oil, alone or in combinations by gavage (5 d/wk) for 14 d [63]	Human PCa 22Rv1 cell-implanted tumor in Nu/J mice	MSA+γT1 or γT2 but not other treatments ↓ tumor weight (∼25%); MSA+γT1 or MSA ↓ PSA
2014	αT or δT at 0.3% diet for 48 d [64]	LNCaP xenograft model in nude mice	δT, but not αT, ↓ LNCaP tumor size and weight, and induced apoptosis in tumors
2016	mTEs containing αTE, βTE, δTE, γTE, and αT (at 8.3, 1.5, 4.6, 11.4, and 6 g out of 100 g, respectively) at 200 or 400 mg/kg BW, by gavage 3 times a week for 8 wk [65]	VCaP human hormone-refractory PCa xenograft model in NCr-immunodeficient mice	The mTEs dose-dependently ↓ tumor growth and caused ↑ CDK inhibitors p21 and p27 and ↑H3K9 acetylation at their promoters with decreased expression of histone deacetylase

γTmT is γT-rich tocopherol mixtures often containing 57%–60% γT, 21%–24% δT, 12%–13% αT, and 0.5%–1.5% βT.

Abbreviations: DMAB, 3,2'-dimethyl-4-aminobiphenyl; HGPIN, high-grade prostatic intraepithelial neoplasia; MNU, N-methyl-N-nitrosourea; Nrf2, nuclear factor erythroid 2–related factor 2; PCa, prostate cancer; PhIP, 2-amino-1-methyl-6-phenylimidazo[4,5-b]pyridine; PIN, prostate intraepithelial neoplasia; mTEs, mixed tocotrienols; TRAMP, transgenic adenocarcinoma of the mouse prostate; TRAP, transgenic rat for adenocarcinoma of prostate; TRF, tocotrienol-rich fraction; ↓, suppress or inhibit; ↑, increase or enhance; ↔, show no effect.

αT and its combination with lycopene have also been assessed in PCa cell-implanted models representing advanced PCa. In the orthotopic human PC-346C prostate xenograft model in NMRI nu/nu mice, combining αT (*all-rac*-α-tocopheryl acetate) and lycopene [5 mg/kg body weight (BW) of each component by oral gavage], not either agent alone, suppressed the growth by 75% of the prostate xenograft and increased median survival by 40% [57]. Interestingly, in Copenhagen rats injected with MatLyLu Dunning PCa cells, dietary αT or lycopene but not their combination increased prostate tumor necrotic areas, whereas none of the treatments significantly affect tumor weight [56]. In addition, αT did not show significant impact on the growth of LNCaP tumors [64].

These preclinical animal studies indicate that αT at relatively high supplement doses do not exhibit preventive effects on early-stage or advanced PCa. Although combining αT with lycopene was shown to block advanced PCa in one animal study, additional research is needed to further test this combination in different PCa models.

#### The effects of αT on PCa in cell studies

Numerous studies have evaluated the impacts of αT and other forms of vitamin E on PCa cells and shown that αT is the least effective among members of the vitamin E family in blocking proliferation of PCa cells, as summarized in Table 3 [55,61,62,64,[66], [67], [68], [69], [70], [71], [72], [73], [74], [75], [76], [77], [78], [79], [80]]. In some studies, αT was found to promote the growth of transformed prostate epithelial cells. Specifically, Njoroge et al. [74] reported that αT promoted the growth of HPV-18 immortalized prostate epithelial (RWPE-1) organoids but did not affect healthy prostate epithelial organoids, whereas decreased malignant LNCaP organoids. Vivarelli et al. [81] showed that *dl*-all-rac-αT at 100 μM, a high pharmaceutical dose, markedly upregulated the expression of phase I CYP enzymes including activators of polycyclic aromatic hydrocarbons and CYP1A1, CYP1A4, CYP2B6, CYP2C9, and CYP4F2 in the RWPE-1 cell line and in rats. Further, αT at this high concentration also enhanced reactive oxygen species and mRNA of cyclooxygenase-2 as well as prostaglandin E_2_, suggesting pro-oxidant and proinflammatory activities. Consistently, supplementation of αT at 100–200 mg/kg BW resulted in increased expression of drug-metabolizing CYP enzymes and oxidative stress markers in rats and even promoted cell transformation frequency, suggesting procarcinogenic potentials [81]. Additionally, αT was recently shown to either counteract or promote immunotherapy. Specifically, αT was shown to increase programmed cell death ligand (PD-L) 1 in PCa DU-145 cells and consistently promote lung tumor development potentially via immunosuppression [78]. In a sharp contrast, potential immunotherapy-promoting effect of αT was suggested in another study where αT at 50 mg/kg enhanced immunotherapy against mammary tumors via reinvigorating dendritic cells [82].

**TABLE 3:** Anti-PCa effects and mechanisms of tocoperols and tocotrienols in cell-based studies

Year and cells	Vitamin E forms	Key finding (IC50s)	Signaling/mechanisms
2002; DU-1145, LNCaP, CaCo-2, SaOs-2 [66]	αT, γT, βT	↓DU-145 cell growth by αT (25–50 μM), γT (12–25 μM), and βT (50 μM)	NA
2004; LNCaP, PC-3, PREC (normal prostate cells) [67]	γT, αT, γT+δT, γT+γTE	↓ LNCaP, PC-3 cell growth by γT (25–50μM) or γT+δT (20+10 μM), γT+γTE (20+2.5 μM), but not αT; ↑ apoptosis in LNCaP cells; no effects on normal cells	↑cytochrome C release, caspase-9 cleavage; ↑ dihydroceramides and dihydrosphingosine, which contributes to anti-PCa
2004; PC-3 [68]	αT, γT, α-CEHC, γ-CEHC, trolox	↓PCa cell growth by γ-CEHC≈γT (<10 μM) > α-CEHC>αT	↓cyclin D1
2004; DU-145, LNCaP [69]	γTE	Combining γTE and statin synergistically ↓ PCa cell growth	NA
2008; LNCaP, PC-3, PZ-HPV-7 [70]	αT, βT, γT, δT, αTE, βTE, γTE, δTE	↓ PCa (but not immortalized cells) growth by δTE (41 μM), γTE (32 μM), βTE (54 μM) >γT, δT>αT, αTE; γTE+docetaxel showed synergy	↑apoptosis, G1 arrest; ↓NF-κB, EGF-R and Id family proteins
2011; LNCaP, PC-3; [61]	γT, γTE	↓ LNCaP, PC-3 cell growth by γTE (10–20 μM); ↑apoptosis, autophagy and necrosis; ↑cytochrome C, PARP cleavage, LC-3; ↓p-AKT after apoptosis started	↑ dihydroceramides and dihydrosphingosine prior to cell death; inhibiting synthesis of sphingolipids counteracted γTE’s anti-PCa
2011; PC-3, DU145 [62]	γTE	γTE (7–12 μM) ↓CD133/CD44 (CSC markers), spheroid formation; CSC-rich population is sensitive to γTE	γTE may target prostate CSCs
2011; PC-3, LNCaP [71]	αT, γT, δT, αTE, γTE, δTE	↓ PC-3 cell growth by δTE (∼23 μM), γTE (30 μM) >>γT, δT>αT, and LNCaP is also sensitive to γT or δT; ↑caspase 3	↑ PPAR-γ, 15-LOX-2 mRNA and protein; ↓TGFβ2, NF-κB and p-p38
2014; LNCaP, VCaP, and CWR22Rv1 [64]	αT, γT, δT, or γ-TmT	δT > γT, γTmT > αT ↓ PCa cell growth, PSA and AR reporter activity; ↑apoptosis	NA
2016; DU145, PC-3, C4-2, LNCaP, CWR22Rv-1 [72]	αT, δT, γT	↓ PCa cell growth, δT (30 μM) > γT (>50 μM) >> αT; δT ↑caspase 3; ↓phosphorylation of AKT (p-AKT)	δT ↓ EGF/IGF-induced p-AKT possibly via blocking EGFR-stimulated PI3K/AKT signaling
2017; LNCaP [73]	γT, δTE	γT+δTE strengthened anti-PCa and enhanced apoptosis	NA
2017; PREG, LNCaP, RWPE-1 [74]	αT	αT (40 μM) ↑ the growth of RWPE-1 organoids, but not healthy prostate epithelial organoids, ↓LNCaP organoids	αT rescue detachment associated decrease in ATP synthesis and fatty acid oxidation in RWPE-1
2019; PC-3, C4-2B [75]	γTE	↓Angiopoietin (Ang)-1 mRNA and protein; ↓CD49f and Bmi-1 (stemness markers); synergy of γTE+Tie-2 inhibitor for ↓cell growth and ↑p-AMPK	γTE+Tie-2 inhibitor may be a novel anti-PCa agent
2019, 2020; PC-3, DU145 [76,77]	δTE	δTE ↑ER stress, autophagy and related p-JNK and p-p38; ↑vacuolation and apoptosis; ↑Ca^2+^ overload in cytosol and mitochondria, ↓mitochondria function	NA
2022; PTEN-Cap8, PC-3, DU145, 22Rv1, LNCaP [55]	αT, γT, δT, γTE, δTE	↓ PCa cell growth by δTE (12 μM) > γTE (17 μM) > αTE (26 μM), δT (24 μM) > γT (49 μM) > αT; ↑apoptosis (caspase 3)	δTE did not show strong inhibition of p-AKT
2022; DU145 [78]	αT	αT (10–30 μM) ↑ cell migration and proliferation, prosurvival signaling, and PD-L1	Upregulation of PD-L1 by αT via activating ERK and STAT3
2022; DU145 [79]	βTE	βTE dose-dependently ↓ PD-L1 and JAK2/STAT3, and T cell–mediated toxicity against DU145	NA
2024; DU145 [80]	αT, γT, δT, βT, αTE, γTE, δTE, βTE	γTE, δTE, βTE dose-dependently ↓ PD-L1; dTE promoted T cell toxicity against DU145	δTE (20 or 30 μM) ↓ glycosylated PD-L1 in cells and exosome

Abbreviations: C4-2B/22RV1, CRPC cells; CRPC, castration-resistant prostate cancer; CSC, cancer stem cell; ER, endoplasmic reticulum; p-JNK, phosphorylated c-Jun N-terminal kinase; PD-L1, programmed death ligand 1; PREG, healthy prostate epithelial cells; prostate cancer cell lines, LNCaP (androgen dependent); PC-3, DU145 (androgen independent); RWPE-1, HPV-18 immortalized prostate epithelial cells; ↓, suppress or inhibit; ↑, increase or enhance; ↔, show no effect.

#### Preclinical cell and animal data support and explain results from large RCTs concerning the effects of high-dose αT supplements on PCa

Preclinical data concerning the impact of αT on PCa (given in previous 2 sections) are largely in agreement with the results from RCTs and may offer explanations of adverse effects observed in the SELECT and HOPE-TOO trials. First, results from RCTs indicate that supplementations of αT at a relatively high dose, that is, 400 IU (364 mg *dl*-αT) or higher, do not offer beneficial effects on PCa prevention among noncurrent smokers. Consistently, αT at 0.05%–0.2% (or higher), which are equivalent to ∼230–920 mg *dl*-αT daily for 60-kg adults, did not show preventive effects on PCa in animal models. Second, preclinical studies showed that αT increased proliferation of transformed prostate epithelial organoids [74] and promoted DNA damage, inflammation, and even benzo[a]pyrene-induced cell transformation [81]. These observations suggest procarcinogenic activities and may explain adverse effects reported in the SELECT where high-dose αT supplementation increased the incidence of early-stage PCa [40]. Third, αT has been reported to elevate the expression of cytochrome P450 enzymes such as CYP3a11 (equivalent to human CYP3A4) via activating pregnane X receptor (PXR) [83,84]. Consistently, αT at 100 μM upregulated several CYP enzymes in human prostate epithelial RWPE-1 cells and in rats. These observations potentially explain side effects of αT in the HOPE/HOPE-TOO trial. Specifically, most participants in the HOPE-TOO trial took multiple medicines including aspirin and β-blockers because of their pre-existing medical conditions. Elevation of drug-metabolizing CYP enzymes by αT (at 400 IU) may counteract therapeutic outcomes of medicines in patients and consequently may contribute to increased hospitalization and rates of heart failure among patients taking αT in the HOPE/HOPE-TOO [37].

In summary, αT at a modest dose (50 mg) is observed to decrease PCa death among heavy smokers. However, supplementation of relatively high doses of αT appears to be ineffective for PCa prevention in RCT trials, which is supported by findings in preclinical cell and animal studies. Preclinical studies also provide rationales for high-dose αT-associated side effects including increased early-stage PCa and hospitalization.

## Chemopreventive and Therapeutic Roles of γT, δT, γTE, and δTE in PCa: Evidence From Preclinical Animal Studies

During the last 20 y, the effects of γT, δT, and γ-tocopherol–rich mixed tocopherols (γTmTs) as well as TEs especially γTE and δTE on PCa development have been investigated in various PCa animal models. These models are believed to recapitulate different-stage PCa development, including formation of PIN, which is precursor lesion of PCa, and progression from PIN to adenocarcinoma [85], as well as the growth of late-stage PCa tumors derived from implanted PCa cells [86]. Consequently, these studies have revealed both preventive and treatment capabilities of these tocopherols and TEs, as summarized in Table 2 [[45], [46], [47], [48], [49], [50], [51], [52], [53], [54], [55], [56], [57], [58], [59], [60], [61], [62], [63], [64], [65]].

### Chemopreventive effects of γT, δT, and γTmT as well as TEs in preclinical PCa models in rodents

Potential chemopreventive abilities of γT, δT, and γTmT, in comparison with αT, have been assessed in preclinical animal models mimicking early-stage PCa development in rodents. For instance, γT was found to inhibit N-methyl-N-nitrosourea plus testosterone-induced PIN in rats’ prostate [50]. In another study, αT, γT, δT, and γTmT are shown to inhibit the number of PINs induced by 2-amino-1-methyl-6-phenylimidazo[4,5-b]pyridine in the CYP1A-humanized mice, and δT was more effective than γT or αT for this effect [51]. Further, in the genetically engineered TRAP and transgenic adenocarcinoma of the mouse prostate (TRAMP) models that spontaneously develop PIN to prostate adenocarcinoma, γT, or γTmT but not αT suppressed prostate adenocarcinoma and decrease palpable tumor incidence, and these anticancer effects were accompanied by increased expression of antioxidant regulator nuclear factor erythroid 2-related factor possibly via modulating DNA methylation [49,52,53]. Interestingly, despite blocking adenocarcinoma, γT did not affect the number of PINs in the TRAP model [49]. In addition, δT but not αT (at 0.2%) has recently been reported to decrease prostate adenocarcinoma but did not have significant impacts on the multiplicity of high-grade PINs in prostate PTEN-knockout mice, whereas αT did not show significant anticancer effects [48]. These observations indicate that γT, δT, and γTmT, but not αT, can inhibit carcinogen-induced hyperplasia and block progression of PIN to prostate adenocarcinoma in spontaneously tumor-developing models.

In addition to tocopherols, TEs have been tested for their chemopreventive effects on PCa in genetically engineered animal models that spontaneously develop PCa. For instance, γTE-rich mixed TEs suppressed prostate tumor development in the TRAMP model [54]. Moreover, δTE (0.05% diet) at a much lowered dosage than δT (0.2% diet) displayed similar inhibitory effects on adenocarcinoma in mice with prostate-specific PTEN knockout [55].

### Anticancer effects of tocopherols and TEs against late-stage PCa in preclinical animal studies

In addition to research on cancer prevention, studies have also been conducted to assess potential treatment effects of tocopherols on late-stage PCa progression in xenograft models bearing implanted PCa cells. Studies assessing potential effects of αT and its combination with lycopene on PCa-implanted tumor development revealed inconsistent results (see “Preventive effects of high doses of aT on PCa in preclinical animal studies” section). In androgen-sensitive human prostate adenocarcinoma LNCaP-implanted xenograft models, γT and δT but not αT modestly suppressed tumor growth [61,64]. γT (∼42 mg/kg BW) or its combined with methaneselenic acid also modestly inhibited the growth of human 22RV1 tumors that represent CRPC [63]. On the contrary, in a study with Dunning R3327H adenocarcinoma rats, which represents slow growing PCa (adenocarcinoma or beyond), γT or its combination with lycopene did not affect tumor growth [59] possibly because of relatively low dose of γT (0.02% diet ≈ 20 mg/kg BW) as at this dose, γT alone did not suppress 22RV1 tumor growth either [63].

The effects of γTE on relatively late-stage PCa have also been assessed. For instance, γTE was found to inhibit tumor development in nude mice implanted with LNCaP xenografts and was stronger than γT for this effect [61]. Furthermore, γTE alone inhibited androgen-independent PC3 prostate tumor growth in nude mice and its combination with docetaxel showed even stronger inhibition of tumor growth [60]. In another xenograft model, mixed TEs in diets inhibited prostate tumor development and increased cyclin-dependent kinase inhibitors p21 and p27 possibly via elevating H3K9 acetylation of their promoters [65]. In addition, TEs have been shown to enhance the efficacy of cancer radiation therapy. Specifically, Kumar et al. [58] reported that γ-irradiation combined with γTE at 400 mg/kg BW (via subcutaneous injection in the neck), but not radiation or γTE alone, reduced the size of established tumors and increased lipid peroxidation in tumors in athymic mice implanted with human PCa PC-3 cells. Moreover, although not specifically related to PCa treatment, γTE and δTE at 200–400 mg/kg BW administered subcutaneously prior to radiation protected hematopoietic stem and progenitor cells in mice after total-body irradiation and accelerated the recovery of white blood cells in irradiated mice [[87], [88], [89], [90]]. These observations suggest that γTE may inhibit advanced PCa tumor progression and potentially be useful for adjuvant therapy for improving treatment efficacy of irradiation and chemotherapy, and γTE and δTE may alleviate irradiation-associated adverse effects.

**FIGURE 3:**
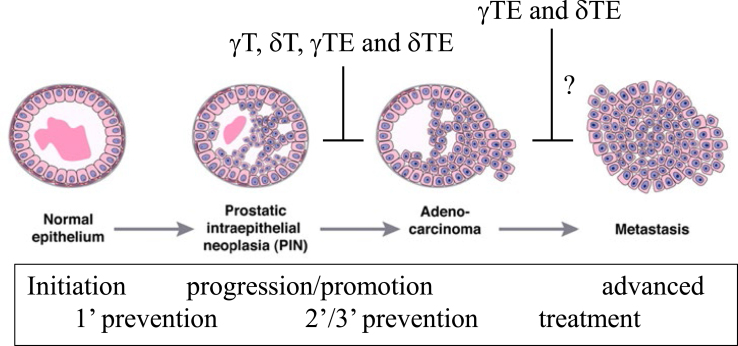
Chemopreventive effects of γT, δT, γTE, and δTE: γT, δT, γTE, and δTE have been shown to block development of prostatic intraepithelial neoplasia (PIN, noncancerous lesion), especially progression from PIN to adenocarcinoma in various PCa animal models. These results suggest that these vitamin E forms may be useful for secondary and tertiary chemoprevention among relatively moderate-risk or high-risk population. This figure is modified based on [85] with permission. 1', 2', and 3' stand for primary, secondary, and tertiary preventions, respectively.

In summary, γT and γT-rich tocopherols, but not αT, are capable of inhibiting carcinogen (N-methyl-N-nitrosourea and 2-amino-1-methyl-6-phenylimidazo[4,5-b]pyridine)-induced prostate epithelial dysplasia. γT, δT, γTE and δTE, although largely not for αT, suppressed progression of PINs to adenocarcinoma in spontaneously tumor-developing models. δTE at a lower dose showed similar anti-PCa efficacy to δT at a much higher dose, indicating stronger chemopreventive effects of δTE (Figure 3) [85]. As to the impact on advanced PCa, γTE is found to be stronger than γT for inhibiting the growth of PCa tumors in xenograft models. Therefore, it can be concluded that γT, δT, γTmT, γTE and δTE, rather than αT, at supplement doses are promising chemopreventive agents for inhibiting relatively early-stage PCa including adenocarcinoma development. Moreover, existing data support that γTE may be useful for treatment or adjuvant therapies for PCa. Despite these data, one noticeable gap is lack of data concerning the effects of δTE on advanced PCa cell growth in animal models. Future studies are needed in this area as δTE appeared to be the strongest among vitamin E forms in blocking PCa cell proliferation in cell-based studies (see further).

## Anticancer Effects of Tocopherols and TEs in PCa Cells

Sustaining cell proliferation and resisting cell death are hallmarks of cancer [91]. Specific vitamin E forms have been shown to inhibit the growth and induce death of PCa cells, as summarized in Table 3 [55,61,62,64,[66], [67], [68], [69], [70], [71], [72], [73], [74], [75], [76], [77], [78], [79], [80]]. In particular, γT and δT but not αT have been shown to inhibit proliferation of PCa cells such as LNCaP, PC-3, and LU145, in a dose-dependent manner, and δT was stronger than γT for blocking PC-3 cell growth [48,67,71]. γT was found to induce apoptosis in LnCaP, but not PC-3 cells, as indicated by DNA fragmentation, increased annexin V translocation to the outside of cytoplasmic membrane, caspase-9 cleavage, and poly (ADP-ribose) polymerase (PARP) cleavage [67]. δT was shown to induce caspase-3 activation and S phase arrest in PC-3 cells and inhibit receptor tyrosine kinase-induced AKT activation [72], although other vitamin E forms including δTE and γTE do not directly target phosphatidylinositol-3-kinase (PI3K)/AKT [48,61]. Compared with tocopherols, TEs displayed stronger anticancer effects in PCa cells. For instance, Jiang et al. [61] showed that γTE is much stronger than γT for blocking PC-3 and LNCaP proliferation and induction of apoptosis and autophagy as well as the growth of LNCaP xenograft tumors. Moreover, δTE was found to be the strongest among all vitamin E forms in inhibition of the growth of PCa cells [55]. Overall, for antiproliferation and induction of apoptosis, γT, δT, γTE, and δTE display stronger anticancer effects than αT, and the relative antiproliferation potency appears to be δTE (IC50 ∼12 μM) ≥ γTE (15–20 μM) > δT (25–40 μM) > γT (30–50 μM) based on the IC50s (parentheses) observed in the aforementioned studies. In contrast, αT has been shown to have no or weak impact on proliferation of cancer cells except for 1 study [66]. These differences among different vitamin E forms were also observed in other type of cancer cells [92].

The mechanisms underlying the differential effects of tocopherols or TEs on PCa cell growth are not fully understood. Nevertheless, as previously discussed [92], differential uptakes of these vitamin E forms into cells may be a factor. For instance, we show that γTE was accumulated at much higher concentrations in human PCa cells than γT, correlating with much stronger anticancer effects of γTE than γT [61]. However, intracellular δT concentrations were found to be comparable with those of αT, but δT was more potent than αT in induction of death in macrophages [93]. Interestingly, γTE and δTE are known to have shorter half-life and lower C_max_ in the plasma than tocopherols in rodents [33] and humans (refer “Different forms of vitamin E and bioavailability” section). Nevertheless, TEs have been found to be accumulated in tumors but not normal tissues in animals [94,95]. In addition, TEs have been proposed to be more uniformly distributed in the membrane bilayer and stronger in disordering of membrane lipids than their tocopherol counterparts [96], which may also contribute to their stronger antiproliferative effects on cancer cells.

## γT, δT, γTE, and δTE as well as Metabolite 13'-COOHs: Molecular Mechanisms Relevant to Prevention and Therapy of PCa

Although all vitamin E forms are considered potent lipophilic antioxidants, the role of antioxidants in cancer prevention is not straightforward and vitamin E forms showed differential anticancer effects in cell and animal models. A reasonable explanation is that different vitamin E forms exert cancer-preventing effects via mechanisms independent of antioxidant activities. Indeed, existing evidence indicates that γT, δT, γTE, and δTE, in contrast to αT, can modulate pathways promoting development and progression of PCa and drug resistance. Moreover, these vitamin E forms, compared with αT, are preferentially metabolized in vivo. 13'-COOHs have been shown to have anticancer and anti-inflammatory effects that may also contribute to anti-PCa effects of the vitamin precursors. In this review, I will focus on the effects and mechanisms of these vitamin E forms and 13'-COOHs on PCa relevant pathways, while anticancer mechanisms relevant to other types of cancer have been reviewed elsewhere [9,92].

### 5-Lipoxygenase is a significant player in PCa development and drug resistance; vitamin E forms and 13'-COOHs block 5-LOX–mediated leukotrienes via distinct mechanisms

5-Lipoxygenase (LOX) is a proinflammatory enzyme catalyzing oxidation of arachidonate to form 5-hydroperoxyeicosatetranoic acid and subsequently leukotriene (LT) A4, which is then converted to LTB_4_ and other LTs [97]. LTB_4_ has been shown to promote PCa [98] and render PCa cells resistance to anoikis [99]. Further, increased 5-LOX expression is found in PCa tissues and associated with worsened prognosis and survival of patients with PCa [100] (The Human Protein Atlas). Consistently, inhibition of 5-LOX has been found to block invasion and induce apoptosis in bone-invading PCa cells [101] and decrease the expression and transcriptional activities of c-*Myc* [102]. Moreover, knockdown or inhibition of 5-LOX has been shown to kill enzalutamide-resistant CRPC cells [101,103]. In addition, inhibition of 5-LOX has also been shown to downregulate PCa stemness and kill PCa stem cells [104].

We demonstrated that tocopherols and TEs block 5-LOX–catalyzed formation of LTB_4_ via different mechanisms. Specifically, γT, δT, γTE, and δTE are much strongly than αT in blocking calcium ionophore-stimulated elevation of LTB_4_ in neutrophils, showing IC50s of ∼4 μM for γTE and δTE, 2–7 μM for δT, 5–10 μM for γT, or >60 μM for αT [105,106]. Mechanistically, γT and δT have been shown to inhibit ionophore-induced Ca^2+^ increase and phosphorylation of JNK (c-Jun N-terminal kinase), the upstream signaling essential for activation of 5-LOX and 5-LOX–mediated LT formation in cells, but these tocopherols do not inhibit human recombinant 5-LOX at physiologically relevant concentrations [105]. γT has also been shown to decrease LTB_4_ or LTC_4_ production in various animal models [107,108]. Unlike tocopherols, γTE and δTE inhibit 5-LOX activity with IC50s of ∼4 μM [106], which is achievable in humans and animals [25,33]. γTE and δTE are stronger than αTE for this activity [106]. Consistent with direct inhibition of 5-LOX, γTE and δTE can block both ionophore-stimulated or thapsigargin-stimulated LTB_4_ in neutrophils but do not affect 5-LOX translocation [106]. Enzyme kinetic studies revealed that γTE and δTE are competitive inhibitors of 5-LOX with Ki of 2.2 μM [106]. These data indicate that γTE and δTE can compete with arachidonate at the active site of the enzyme.

In addition to the vitamin precursors, we demonstrated that 13'-COOHs are strong inhibitors of 5-LOX with IC50s ranging from 0.1 to 1 μM. Specifically, δT-13'-COOH, a metabolite from δT, can inhibit human 5-LOX activity with IC50 of ∼1 μM [105]. δTE-13'-COOH, a metabolite of δTE, more strongly inhibits 5-LOX (IC50 ∼0.6 μM) than its precursor [106,109]. Consistently, Pein et al. [110] reported that αT-13'-COOH, a metabolite from αT, and δTE-13'-COOH (purified from *Garcinia kola*) inhibit 5-LOX with IC50s ∼0.1 μM and decreased LTs in vivo [110]. In addition, our enzyme kinetic data showed that δT-13'-COOH and δTE-13'-COOH are competitive inhibitors of human 5-LOX with Ki of 1.6 and 0.8 μM, respectively [106].

### γTE and δTE can block nuclear factor-κB that plays significant roles in PCa

Nuclear factor (NF)-κB is a transcriptional factor regulating prosurvival and proinflammatory genes. In particular, it has been shown that NF-κB is the third most activated pathway out of >100 pathways in metastatic and primary PCa where it correlates with disease progression, poor prognosis, and treatment resistance [[111], [112], [113]]. In particular, NF-κB signaling and its target genes are upregulated in CRPC tumors and associated with shorter time to death from disease recurrence [113]. Furthermore, NF-κB activation contributes to development of CRPC and resistance to enzalutamide, an androgen receptor antagonist, or chemotherapy drug docetaxel, and inhibition of NF-κB restored responsiveness to these treatments [114,115].

We have shown that γTE and δTE can inhibit TNFα-stimulated NF-κB activation via induction of A20. Specifically, δTE and γTE are stronger than tocopherols (γT, δT, or αT) and δTE is stronger than γTE in blocking TNF-α–stimulated phosphorylation of IκBa and nuclear translocation of P65 in macrophages and cancer cells including PCa PC-3 cells [116,117]. Mechanistic studies revealed that δTE and γTE inhibited NF-κB via induction of A20, which is also known as TNF-α–induced protein 3 and an endogenous negative regulator of NF-κB [118]. In particular, γTE and δTE treatment elevated A20 mRNA and protein in macrophages and PC-3 cells [116,117]. In A20^−/−^ cells, anti–NF-κB by these compounds diminished compared with that in A20^+/+^ cells, supporting a causative role of A20 upregulation in blocking activation of NF-κB [116,117]. Further research indicates that induction of A20 was caused by modulation of sphingolipids by these TEs, including accumulation of dihydroceramides and dihydrosphingosine [116,117]. Consistent with these mechanistic studies in cells, γTE and δTE have been shown to inhibit NF-κB in preclinical cancer models [119,120].

### Cholesterol synthesis, HMG-CoA reductase, and CRPC progression

Cholesterol is essential in maintaining membrane physical properties, and its metabolism is involved in steroid hormone biosynthesis. Cancer cells rely on cholesterol to satisfy their increased nutrient demands and support their uncontrolled growth, thus promoting tumor development and progression [121]. Advanced PCa including CRPC is shown to express increased HMG-CoA reductase (HMGCR), the rate-limiting enzyme for cholesterol biosynthesis [122], and have higher cholesterol ester concentrations than normal cells, which may play a role in intratumoral steroidogenesis in CRPCs [123]. Moreover, increasing evidence supports an inverse association between the use of statin (HMGCR inhibitor) and advanced PCa risk, although its association with general PCa risk is not consistent [124]. This observation supports the importance of steroidogenesis in advanced PCa. Besides steroidogenesis, excessive lipids and cholesterol in cancer cells appear to be stored in lipid droplets, and intratumor cholesteryl ester accumulation is associated with cancer aggressiveness and recurrence [[125], [126], [127]]. Consistently, simvastatin, a potent inhibitor of HMGCR, promoted apoptosis in PCa cells, and this anticancer effect was reversed by replenishing cell membrane with cholesterol [128]. Additionally, knockdown of HMGCR or statin sensitized drug-resistant CRPC cells and tumors [122]. These results suggest that HMGCR may be a target of advanced PCa. Interestingly, δTE and γTE but not tocopherols have been shown to induce degradation of HMGCR in liver cells [129], and combining γTE or δTE with statin synergistically inhibited the growth of many types of cancer cells including PCa DU-145 cells [130]. However, the underlying mechanism of such synergy remains to be determined including whether these TEs can degrade HMGCR in PCa cells, potential contributions from other activities by TEs (e.g., blocking 5-LOX), and the possibility that γTE can reverse statin-induced increase of HMGCR [131]. It is also not clear whether combining γTE and δTE with statin could synergistically block PCa in vivo.

### Anti-PCa cells via modulation of sphingolipids

γT and γTE have been shown to induce apoptosis and inhibit proliferation of PCa and other cancer cells at least in part by modulating sphingolipids [9,92,132]. Sphingolipids such as dihydroceramides, dihydrosphoingosine, and ceramides have been recognized to play important roles in regulating cell death and survival, as elevation of these sphingolipids has been shown to cause stress, inhibit cell growth, and induce apoptosis [[133], [134], [135]]. To this end, γT, γTE, and 13'-COOHs have been shown to elevate dihydrosphingosine and dihydroceramides as well as ceramides in prostate and other types of cells. Importantly, the modulation of these sphingolipids has been observed prior to manifestation of cell death such as PARP cleavage [109,132,136]. This is important for supporting the notion that modulation of sphingolipids may be the cause instead of the consequence of cell death. Mechanistic studies have revealed that γTE and 13'-COOHs inhibited the activity of dihydroceramide desaturase, a key enzyme in the de novo synthesis of sphingolipids pathway [109,136]. Prolonged treatment with γTE or 13'-COOHs are found to elevate ceramides possibly via activation of sphingomyelinase [109,136]. Consistent with the idea that modulation of sphingolipids contributes to induction of death, pharmalogic inhibition of the de novo synthesis of sphingolipids partially counteracted γT-induced or γTE-induced anticancer effects in cancer cells [67,136]. These observations support molecular interaction between these compounds and sphingolipids, although whether these vitamin E forms can modulate sphingolipids in vivo remains to be investigated.

### Additional mechanisms: PI3K/AKT, angiogenesis, cancer stem cell, and epigenetic modulation as well as potential implications in immunotherapy

PI3K/AKT signaling is believed to contribute to PCa [137]. Potential impact of vitamin E forms on PI3K/AKT signaling has been investigated in PCa cells. For instance, γTE was found to reduce phosphorylation of AKT (p-AKT), a key target of PI3K, after apoptosis induced by γTE was evident [61]. This observation suggests that inhibition of PI3K/AKT is not likely a cause of death induction by γTE. Similarly, δTE did not appear to strongly inhibit PI3K/AKT in PCa cells [55]. On the contrary, δT was found to block EGF/IGF-induced phosphorylation of AKT in PCa cells [72]. Interestingly, stably expressing dominant active AKT or PI3K resulted in diminished anticancer effects by δT, supporting PI3K/AKT as a target of δT [72]. Consistently, δT also decreased AKT phosphorylation in the prostate in Pten^P−/−^ mice [48]. These results indicate that δT, but no other vitamin E forms, can target PI3K/AKT pathway.

Angiogenesis is a hallmark of cancer [91]. Hypoxia-inducible factor (HIF)-1α, and its downstream genes like vascular endothelial growth factor (VEGF), IL-8, and cyclooxygenase 2 play critical roles in neovascularization. Interestingly, γTE and δTE have been shown to be stronger than αTE or βTE in inhibition of hypoxia-induced VEGF mRNA and its secretion. δTE at 2 μM was also reported to reduce HIF-1α protein expression and/or increase its degradation and decrease IL-8 [138]. In addition, γTE was shown to suppress angiopoietin (Ang-1)/Tie-2, a well-recognized signaling pathway involved in regulation of angiogenesis [75,139]. These observations suggest that δTE and γTE may exert anticancer effects via blocking angiogenesis, although it remains to be determined whether this mechanism plays a role in inhibition of PCa in vivo.

γTE may target cancer stem cells (CSCs) that are recognized to contribute to resistance to cancer therapy [140]. Specifically, γTE has been shown to downregulate the expression of CD133/CD44, CSC markers, in androgen-independent PCa cells. Consistently, γTE inhibited the formation of spheroids [62]. Interestingly, although CSC-enriched PC-3 cells (CD133+) are resistant to docetaxel, these cells and CD133-negative cells appeared to be sensitive to γTE treatment [62]. Given that blocking 5-LOX has been shown to decrease PCa stemness and kill CSCs [104], γTE’s antistemness effect may be partially rooted in its inhibition of 5-LOX [106].

Evidence suggests that vitamin E forms may be capable of modulation of gene expression via epigenetic mechanisms. In particular, γT-rich mixed tocopherols have been shown to decrease the expression of DNA methyltransferases (DNMTs) including DNMT1, DNMT3A, and DNMT3B in the prostate of mice in the TRAMP model [53], suggesting the possibility of modulating DNA methylation and consequently gene expression. Consistently, in the TRAMP model, γT-rich mixed tocopherols were found to inhibit CpG methylation in the promoter of nuclear factor erythroid 2–related factor 2 compared with controls [53]. In addition, a TE mixture was reported to induce epigenetic modification including acetylation of cyclin dependent kinases (CDK) inhibitors p21 and p27 in mice [65]. Despite these observations, the mechanism underlying decreased expression of DNMT or modulating acetylation by γTmT remains to be determined.

Recently, vitamin E forms have been reported to potentially influence immunotherapy via immunomodulatory activities. To this end, contradictory roles of αT in immunotherapy have been reported. On the one hand, αT was found to increase PD-L1 in PCa DU-145 cells, which may contribute to αT-associated promotion of tumorigenesis [78]. On the other hand, αT was also shown to enhance immunotherapy against mammary tumors via reinvigorating dendritic cells [82]. Recently, βTE and δTE have been shown to dose-dependently suppress the expression of PD-L1 in PCa DU145 cells and enhance T cell–mediated toxicity against these cells [79,80]. Further mechanistic investigations show that treatment of δTE (20–30 μM) reduced glycosylated PD-L1 [80], which is essential for interaction between PD-L1 and programmed cell death protein 1 and therefore immunosuppression [141,142]. δTE treatment also decreased exosomal PD-L1 and glycosylated PD-L1 [80].

**FIGURE 4:**
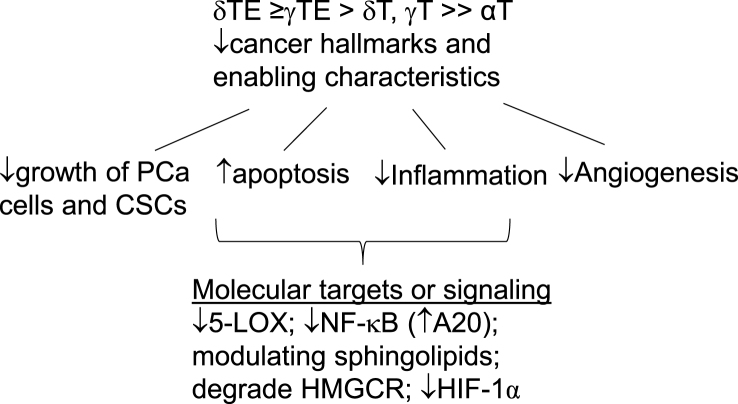
Anti-PCa effects and mechanisms of vitamin E forms: γT, δT, γTE, and δTE inhibit several cancer hallmarks (uncontrolled proliferation, evading apoptosis, and angiogenesis) and enabling characteristics (inflammation) via targeting 5-LOX, NF-κB, sphingolipids, and HMGCR. 5-LOX, 5-lipoxygenase; HMGCR, HMG-CoA reductase.

In summary, accumulating research has indicated that γT, δT, γTE, and δTE can inhibit cancer hallmarks and enabling characteristics including uncontrolled cell proliferation, resistance to apoptosis, angiogenesis, and inflammation by blocking PCa-promoting signaling including NF-κB, 5-LOX, sphingolipids, and possibly cholesterol synthesis as well as epigenetic modulation (Figure 4). Emerging evidence suggests potential roles of vitamin E forms in immunotherapy, an area warranting further investigation.

## The Impact of Vitamin E Forms and 13'-COOHs on PXR and CYP Enzymes: Relevance to PCa and Drug Interactions

PXR is an adopted orphan unclear receptor and plays a major role in the metabolism and clearance of drugs in the liver and intestine via induction of drug-metabolizing enzymes such as CYPs and transporting proteins including intestinal P-glycoprotein (P-gp), a member of the ATP (adnosine triphospate) binding cassette transport family (ABC transporter) [143,144]. In addition to regulation of drug metabolism, the role of PXR in cancer including PCa has also been extensively studied and believed to be context specific as evidence supporting pro-PCa and anti-PCa roles of PXR has been documented [143]. On the one hand, increased expression of PXR has been observed in PCa tissues [145]. On the other hand, higher PXR expression and its targeted gene *CYP3A4* appear to correlate with favorable prognosis and increased survival in PCa patients [146]. Consistently, reduced PXR was found to correlate with a loss of a tumor suppressor protein in CRPC [147].

Vitamin E forms and 13'-COOHs have been shown to differentially affect PXR and CYPs. For instance, αT but not γT or γTE-induced CYP3a11 (the murine homolog to human CYP3A4) in mice, whereas αTE and γTE were stronger than αT in induction of a report gene driven by PXR [83]. Further, αT supplementation activated PXR in human leukocytes [84] and increased many drug-metabolizing enzyme genes in prostate epithelial cells [81]. αTE, γTE, δTE, and βTE but not tocopherols were found to activate PXR in HepG2 cells [148]. In addition to the vitamers, 13'-COOHs appear to be potent activators of PXR. αT-13'-COOH and γTE have been shown to increase PXR activity and its regulated P-gp expression [149]. 13'-COOHs including δTE-13'-COOH are shown to be agonist of PXR with EC50s of 1.5–3.3 μM and mice treated with δTE-13'-COOH had increased expression of PXR, CYP3A4 and multidrug resistance protein (MDR1) [150]. These observations indicate that TEs, αT and 13'-COOHs are capable of modulating drug metabolism via interacting with PXR, suggesting their potential impacts on drug metabolism. Therefore, activation of PXR and regulated drug-metabolizing enzymes should be considered when combinatory drugs are developed. In addition, given that PXR may play context specific roles in PCa, interacting with PXR and its regulated genes may be a mechanism underlying modulatory effects of vitamin E forms on PCa.

## The Effects of γT-Rich Tocopherols on PCa and TEs on Other Types of Cancer in Human Clinical Studies

Although existing preclinical evidence supports that non-αT vitamin E forms are promising for PCa prevention, there is only 1 publication so far documenting supplementation of γT-rich mixed tocopherols in patients with PCa in a phase 0 trial (NCT00895115). Specifically, 59 men diagnosed with localized PCa were randomized to receive no supplementation or daily supplements of γTmT (containing αT, γT, and δT at 128, 200, and 71 mg twice a day, respectively) for 1 wk, or the supplements for 2 wk, before undergoing radical prostatectomy. Upon completing the study, Goodin et al. [23] reported that although there were no significant changes of tocopherols in the fasting plasma, patients taking γTmT supplements had significantly increased γT and δT in the prostate tissue after 2 wk of supplementation. Meanwhile, supplementation of γTmT resulted in elevation of γ-CEHC, δ-CEHC, and 5'-COOHs in the plasma and prostate as well as urine [23]. In additional to these bioavailability results, supplementation of γTmT was found to be safe and resulted in a nonsignificant decrease of PSA [23].

Despite scarcity of human studies on PCa, the use of TEs and δTE as adjuvants for chemotherapies have been tested in several intervention trials for treating pancreatic, breast, colorectal, and cervical cancers. Specifically, in a double-blinded controlled clinical trial, a combination of TE-rich fraction and tamoxifen did not significantly improve breast cancer specific survival rate compared with tamoxifen-placebo controls [151]. Similarly, Kjaer et al. [152] reported that δTE at 300 mg thrice a day did not enhance the efficacy of neoadjuvant breast cancer treatment or reduce the frequency of side effects in a phase II trial involving 80 patients with newly diagnosed breast cancer (NCT02909751). On the contrary, δTE at daily 400–1600 mg induced apoptosis in dysplastic or malignant pancreatic tissues in a phase I trial including 25 patients for curative surgical resection with presumptive premalignant or malignant neoplasms of exocrine pancreas (NCT00985777) [25]. In a randomized, double-blind, placebo-controlled phase II study, the use of δTE (300 mg thrice a day) as an adjuvant for FOLFOXIRI (5-fluorouracid, oxaliplatin, and irinotecan) was assessed in patients with metastatic colorectal cancer. It was concluded that δTE treatment, although did not improve efficacy of FOLFOXIRI, resulted in dose reduction of oxaliplatin, suggesting a possible neuroprotective effect of δTE [153]. Interestingly, in a phase II trial including 23 patients diagnosed with stage III platinum-resistant ovarian cancer (NCT02399592), treatment with δTE (300 mg thrice daily) and bevacizumab resulted in 70% disease control [154], which appears to be better than bevacizumab alone [155]. Together, these studies suggest that potential use of δTE as adjuvant therapies for improving treatment outcomes should be further explored for treating ovarian and pancreatic cancers.

## Conclusions and Future Directions

From 1980s to early 2010s, research of vitamin E intervention against PCa was focused on αT. The ATBC trial reported that modest αT supplement (50 mg) decreased PCa death among heavy smokers. This observation encouraged subsequent RCTs including the SELECT, which, however, observed that αT at ≥400 IU show no beneficial effects or even increased risk of early-stage PCa incidence among nonsmokers. Interestingly, animal and cell-based studies also failed to show beneficial effects of high doses of αT on PCa. In another word, the lack of PCa-preventive effects of high-dose αT supplement in preclinical studies is in agreement with no beneficial effects observed in RCTs. Therefore, preclinical and clinical data indicate that αT at relatively high doses will not likely prevent early-stage PCa development in nonsmokers but may exert adverse effects via induction of drug-metabolizing enzymes and even pro-oxidant effects. As to moderate supplementation of αT, it is encouraging that αT at 50 mg decreased PCa-associated death in heavy smokers in the ATBC trail, but this beneficial effect should be further assessed. This is because PCa was a secondary end point and there was no pretrial screening of PSA or DRE in the ATBC trial, and therefore, the observed beneficial effects of αT may be accidental. Nevertheless, given that smoking is still relatively prevalent, potential benefit of αT to prevention of clinical PCa among smokers is relevant to public health and deserves further investigation. It is this author’s opinion that preclinical research in animal models should be conducted to test the idea that modest αT supplement may prevent PCa under high oxidative stress conditions such as smoking. On confirmation with relevant preclinical work, the effects of αT on PCa should be further assessed in a RCT where the effect of αT at modest doses (50–100 mg) on clinical PCa (as the primary end point) should be examined among smokers.

Starting from 2002, traditionally ignored vitamin E forms have been researched on their ability to modulate PCa, including γT, δT, and TEs (mainly γTE and δTE) in preclinical cell and animal studies. In particular, these vitamin E forms show much stronger anti-PCa effects than αT in cell studies, with relative potency concerning antiproliferation and proapoptosis of δTE, γTE > δT > γT >> αT. Further, γT, γTmT, and δT have been found to be effective for inhibiting relatively early-stage PCa, as indicated in carcinogens-induced and genetically engineered PCa models, whereas display modest effects against late-stage cancer as indicated in PCa cell-implanted xenograft models. Meanwhile, γTE and δTE are shown to strongly inhibit development of prostate adenocarcinoma in genetically engineered spontaneously tumor-developing models. For this effect, δTE appears to be stronger than δT, the latter being more effective than γT. Additionally, amber evidence also suggests that γTE can block implanted PCa cells associated tumor development in xenograft models and is stronger than γT for this effect. γTE can also enhance anti-PCa effects of chemotherapeutic drugs in mice. Overall, these anti-PCa effects of γT, δTE, γTE, and δTE observed in preclinical studies provide evidence supporting the notion that these compounds may be useful for preventing early-stage PCa progression in people at moderate-risk and high-risk of PCa. Considering their good safety, these compounds should be considered in RCT trials for secondary and tertiary (especially γTE and δTE) chemoprevention.

Despite promising data supporting chemopreventive and even therapeutic effects of γT, δT, γTE, and δTE against PCa in preclinical research, future research is needed to further assess their anti-PCa efficacy and elucidate underlying mechanisms. First, among different forms of vitamin E, δTE appears to show the strongest antiproliferative effect against advanced PCa cells, but there is lack of documentation of the impact of δTE on PCa tumor development in xenograft models. This is a significant gap because such evidence is essential for recommending the use of δTE for inhibiting relatively late-stage PCa including CRPC in RCTs. Therefore, the effectiveness of δTE for blocking PCa progression or recurrence should be assessed in PCa-implanted models including patient-derived xenograft models. Second, potential use of γTE or δTE as an adjuvant for improving therapeutic outcomes and possibly offering neuroprotection of chemotherapy or radiation therapy are supported by preclinical research [58,[87], [88], [89], [90]] and some encouraging observations of δTE as an adjuvant therapy for ovarian and colorectal cancers in RCTs [153,154]. Therefore, the anticancer efficacy of δTE in combinations with therapeutic agents should also be tested using PCa cell-implanted and patient-derived xenograft models. Third, the gut microbiota have been shown to contribute to endocrine resistance in CRPC by providing an alternative source of androgens [156]. Interestingly, γT and δTE/γTE as well as δTE-13'-COOH have been reported to cause favorable changes of gut microbiome in cancer models [157,158] or under proinflammatory conditions [34,159]. In particular, δTE/γTE (8:1) has been found to significantly increase [*Eubacerium*] *coprostanoligenes* and *Parabacteroides goldsteinii* CL02T12C30, which are known to be involved in the metabolism of cholesterol, the precursor of androgen [158]. It is therefore of importance to assessing potential modulation of gut microbes by these vitamin E forms in PCa models and evaluating the role of modulating gut microbiome in anti-PCa by different vitamin E forms. Finally, γT, δT, γTE, and δTE are readily metabolized in vivo. Although anticancer effects of their metabolites including CEHCs and 13'-COOHs have been observed in cell-based studies, potential impacts of 13'-COOH on PCa cells have not been assessed [26,68,160]. Therefore, the effects of 13'-COOHs on the growth of PCa cells should be evaluated in vitro and in vivo, and such research will offer mechanistic insights into the role of metabolites in anti-PCa effects by precursor vitamin E forms.

## Authors contributions

The sole author had responsibility for all aspects of the manuscript. During the preparation of this work, the author did not use generative AI and AI-assisted technologies and takes full responsibility for the content of the publication.

## Conflict of interest

The author reports no conflict of interest.
